# Sequence analysis of feline immunoglobulin mRNAs and the development of a felinized monoclonal antibody specific to feline panleukopenia virus

**DOI:** 10.1038/s41598-017-12725-5

**Published:** 2017-10-05

**Authors:** Zhengchun Lu, Rebecca L. Tallmadge, Heather M. Callaway, M. Julia B. Felippe, John S. L. Parker

**Affiliations:** 1 Baker Institute for Animal Health, College of Veterinary Medicine, Cornell University, thaca, NY 14853 USA; 2 Department of Clinical Sciences, College of Veterinary Medicine, Cornell University, Ithaca, NY 14853 USA

## Abstract

In response to immunization, B-cells generate a repertoire of antigen-specific antibodies. Antibody-based immunotherapies hold great promise for treating a variety of diseases in humans. Application of antibody-based immunotherapy in cats is limited by the lack of species-specific complete sequences for mRNAs encoding rearranged heavy and light chain immunoglobulins in B cells. To address this barrier, we isolated mRNAs from feline peripheral blood mononuclear cells (PBMCs), and used available immunoglobulin sequences and 5′ and 3′ RACE to clone and sequence heavy and light chain immunoglobulin mRNAs. We recovered mRNA from PBMCs from two cats, cloned and sequenced the variable and constant domains of the feline heavy chains of IgG1a (IGHG1a), IgG2 (IGHG2), and IgA (IGHA), and the light chains (lambda and kappa). Using these sequences, we prepared two bicistronic vectors for mammalian expression of a representative feline heavy (IGHG1a) together with a light (lambda or kappa) chain. Here we report novel feline Ig sequences, a technique to express antigen-specific felinized monoclonal antibodies, and the initial characterization of a functional felinized monoclonal antibody against feline panleukopenia virus.

## Introduction

Adaptive humoral immunity protects vertebrates from pathogens by generating a repertoire of antigen-specific immunoglobulins (Igs) or antibodies. Antibodies are produced by plasma cells derived from B-cells and, in humans, the B-cell repertoire has been defined by cloning and sequencing the cognate immunoglobulin variable heavy (IGH) and light lambda (IGL) or kappa (IGK) chains displayed on memory B-cells and antibody-secreting plasma cells^1,2^. Other studies have identified rare broadly neutralizing monoclonal antibodies (mAbs) against important viral pathogens such as HIV, dengue and influenza A viruses, and have allowed the identification of therapeutic targets for autoimmune diseases and cancer^3–8^. In addition, next generation sequencing of B-cells from individual patients has defined the cognate Ig heavy and light variable domains of over 10^6^ clones^9,10^. In contrast, little to no information is available about the diversity of the feline IgG response to viruses, and limited sequence information is available for feline Ig mRNAs in general.

Ig molecules consist of four polypeptide chains: two identical heavy chains and two identical light chains that are linked by disulfide bonding. The light and heavy Ig chains fold into domains that have been defined based on their sequence conservation as constant and variable. There are two identical antigen recognition sites per Ig and they are formed by the paired variable domains of a Ig heavy and light chain. Each structure of the variable domain of the heavy and light chains is an anti-parallel β-sheet sandwich consisting of nine β-sheets linked by loops. Six hypervariable loop regions called complementarity-determining regions (CDRs) make direct contact with the epitope of antigens. The variable heavy and light chain of each arm of an antibody contribute with three CDRs each to the antigen-recognition site or paratope. The CDR loops are connected by less variable β-sheet framework (FR) regions^11^.

The mRNA encoding individual heavy or light chains results from DNA recombination of gene segments that encode constant, variable, joining and diversity genes^12^. The variable domain of the Ig heavy (IGH) chain is encoded by three different gene loci: the variable (IGHV) gene, a diversity (D) gene and a joining (J) gene^13^. The variable domains of IGL or IGK chains are encoded by two gene segments: variable (IGLV, IGKV) and joining (J) genes. The recombination of these gene segments (multiple variable, fewer diversity and some joining genes) by DNA rearrangement results in the combinatorial diversity of Ig heavy and Ig light chains. This is the primary mechanism responsible for antibody repertoire diversity^14^.

In most species, the IGH variable locus contains multiple IGHV genes. Most IGHV genes are functional and able to encode proteins, but some are pseudogenes and are noncoding. The total number of IGHV genes varies by species: there are over 160 in mice and rats, 100 in human, 80 in dogs, 50 in horses, and less than 20 in cows and sheep. In cats, 64 IGHV genes with 42 functional genes and 22 pseudogenes have been predicted from an early assembly of the feline genome sequence^15^. The full-length sequences of feline mRNAs encoding immunoglobulin heavy and light chains have not been completely characterized; thus, neither the complement of germline Ig heavy, lambda, or kappa variable genes that are expressed (also referred to as “gene usage”) in the feline Ig repertoire is known nor the extent of nucleotide diversity from the germline sequence. Two subclasses of the feline IgG constant domain are described, IgG1 and IgG2, with IgG1 being the predominant subclass (~98%)^16,17^. Two alleles of the feline IGHG1 heavy chain gene (Cγ1^a^ and Cγ1^b^) encode IgG heavy chain 1a and 1b proteins, and the usage frequency of each gene has been reported to be approximately 62% and 36%, respectively^16^. The CDRs of 24 feline IGHG variable domains have been sequenced and show structural homology with CDRs of human Ig mRNAs^18^. Information about the feline Ig light chain is limited. Cats appear to express the lambda light chain primarily, and the expressed protein ratio of lambda to kappa chain that is reported varies from 3:1 to 92:8^19–21^. Only two feline Ig light chain sequences are publicly available: one partial Ig lambda constant domain sequence and one full-length Ig kappa sequence.

To better understand the antibody repertoire in cats, detailed knowledge of the germline Ig gene usage and full-length sequences of heavy and light chain mRNAs are necessary. Here we identify mRNA sequences for feline IgG heavy and light chains. The sequences of the variable and constant domains of the feline IgG heavy chain (IGHG1a and IGHG2), light chain (lambda and kappa) as well as constant domain of IgA were obtained. The expressed IGH (variable and constant), IGL (variable and constant) and IGK (variable and constant) genes were mapped to feline chromosomes B3, D3, and A3, respectively. We subsequently expressed feline Ig in a bicistronic mammalian vector system using representative sequences of heavy and light chain. The expressed feline antibodies assembled correctly and we used the vector to generate a ‘felinized’ mAb by swapping the CDRs 1–3 derived from the heavy and light chains of a feline panleukopenia virus-specific rat mAb (mAb E) with those present in the parental feline Ig expression vector. CPV and feline panleukopenia virus (FPV) are classified as host range variants of feline parvovirus in the genus of *Parvovirus*, both of which share >99% identical nucleotide sequences and showed similar antigenicity^22–24^. Neutralizing mAb E was originally derived against FPV and is well characterized by its nucleotide sequences and structural interactions with CPV^25^. The engineered ‘felinized’ mAb E maintained its antigen recognition capacity and was functional in hemagluttination inhibition assays.

## Material and Methods

### Sample collection and preparation

Two healthy domestic short hair cats (male Cat#1 and female Cat#2,10- and 6-month-old at the time of blood collection, respectively) were included in this study. Both cats were vaccinated with Fel-O-Vax^®^ (Boehringer Ingelheim Vetmedica, Duluth, GA). Six to eight ml of heparinized peripheral blood from each cat was purchased from Liberty Research, Inc (Waverly, NY). All blood collections were carried out in accordance with IACUC guidelines (IACUC Protocol 14.1800.012: Maintenance of Colony Felines for Sale or Transfer to Research Protocols) and under the United States Department of Agriculture (USDA) Animal Welfare Act regulations (Certificate No. 21-A-0047). Peripheral blood mononuclear cells (PBMCs) were harvested by density gradient centrifugation as described^26^. In brief, blood was diluted 1:1 with PBS, drop-wise layered onto Histopaque-1077 (Sigma-Aldrich, St. Louis, MO), and centrifuged at 400 × *g* for 30 min at room temperature. The PBMC layer at the interface between the plasma and gradient solution layers was collected and washed 3 times with PBS. Total RNA was extracted from the PBMCs using a Qiagen RNeasy kit (Qiagen, Valencia, CA). RNA quality was assessed by bioanalyzer (Agilent technology, Santa Clara, CA).

### Primer design

Primers to generate RACE libraries were designed using feline Ig sequences obtained from Genbank or from RACE sequences generated during this project using Primer 3 (Supplementary Table S1)^27^. Sequence (GATTACGCCAAGCTT) that overlaps with the RACE vector was added at the 5′ end of each RACE primer to facilitate subsequent cloning.

### RACE library construction

Total RNA extracted from cat PBMCs was used to synthesize 5′ or 3′ RACE-ready cDNA using the SMARTer^®^ RACE 5′/3′ kit (Takara Bio USA, Mountain View, CA). PCR amplification of different Ig fragments (IGHV, IGHC, IGLV, IGLC and full-length IGK) was performed using gene-specific primers together with a universal primer mix provided by the manufacturer (UPM, Supplementary Table S1). PCR reactions contained 5′ or 3′ RACE-Ready cDNA, 1× iProof high fidelity buffer (HF), 0.2 mM dNTPs, 0.5 μM gene-specific primer, 1× UPM, and 0.02 U iProof DNA polymerase (Bio-Rad Laboratories, Hercules, CA). Touch down PCR was used in most PCR amplifications. Thermal cycling parameters were 5 cycles of 98 °C for 30 s and 72 °C for 1 min; 5 cycles of 98 °C for 30 s; 70 °C for 30 s and 72 °C for 1 min; and 25 cycles of 98 °C for 30 s, 64–68 °C for 30 s and 72 °C for 1 min. Regular PCR cycling conditions were used when touchdown PCR failed to amplify the IGHC domain. In such cases, 1× iProof GC buffer was used to replace the 1× iProof HF buffer. Thermal cycling parameters were 98 °C for 30 s; 35 cycles of 98 °C for 30 s, 66 °C for 30 s, 72 °C for 1 min; final extension was 72 °C for 10 min. PCR products were gel purified using Wizard^®^ SV and PCR Clean-up System (Promega, Madison, WI), and cloned into linearized pRACE-vector with In-Fusion HD master mix, both of which were provided by the SMARTer kit. Individual clones with ampicillin resistant genes were selected and plasmid DNA was purified with GenCatch™ plasmid DNA miniprep kit (Epoch Life Science, Missouri City, Texas).

### Sequence analysis

Clones were Sanger sequenced with M13F (5′-CCCAGTCACACGTTGTAAAACG-3′) and M13R (5′-AGCGGATAACAATTTCACACAGG-3′) primers at the Cornell University Institute of Biotechnology Genomics Facility, Ithaca, NY. Internal sequencing primers for IGHG (5′-CAGTTCAACAGCACCTACCG-3′) and IGHA (5′-TGTGTCCAGTGTCCTTCCAG-3′) were also used to cover the complete constant domain of IGHG or IGHA. Replicate clone sequences and sequences with internal deletions or premature stop codons were eliminated from the analysis. Sequences are available through Genbank with accession numbers KY795030-KY795318.

The annotation of framework (FR) and complementarity-determining regions (CDRs) of each clone sequence was determined using the ImMunoGeneTics (IMGT) database (http://www.imgt.org/IMGTrepertoire/LocusGenes/#F)^28^. IMGT guidelines for Ig nomenclature were used throughout. The index of variability of amino acid polymorphism in CDR3 was calculated by dividing the number of different amino acids at a given residue by the frequency of the most common amino acid at that residue^29^.

Percentage of germline identity was determined by comparison of clone nucleotide sequences to the *Felis catus* reference genome (assembly Felis_catus_8.0). Gene usage was determined by mapping clone sequences to the feline reference chromosomes using NCBI BLAST and Splign alignment tools (NC_018728 for IGH clones; NC_018734 for IGL clones; NC_018725.2 for IGK clones) (https://www.ncbi.nlm.nih.gov/sutils/splign/splign.cgi).

### Phylogenetic analysis

Phylogenetic analysis of 71 clone sequences of IGHV obtained from 2 cats was conducted using MEGA7^30^ software. The Maximum Likelihood method on the Tamura-Nei model was used for the analysis. The phylogenetic tree was illustrated using FigTree (version 1.4.3).

### Construction of expression vectors by Gibson assembly

The vector pVITRO-dV-IgG1/κ^31^ (Addgene, Cambridge, MA) was used as a backbone to generate the feline Ig expression vector. Three representative sequences of feline IGHG1a, IGL and IGK from our RACE libraries were synthesized with overhang sequences that matched the flanking regions of the IgG1 and IgK chain of the pVITRO-dV-IgG1/κ. A Kozak consensus sequence (CACC) was added before the start codon of all three PCR fragments. The linear pVITRO-dV-IgG1/κ backbone was amplified using primers located at the flanking region of human IgG1 or human κ. Inserts and vector backbone fragments were joined by Gibson assembly (Gibson Assembly Kit; New England Biolabs) to generate plasmids pVITRO-cat IgG-IgL and pVITRO-Cat IgG-IgK. All plasmid sequences were verified.

A similar Gibson cloning strategy was used to swap the CDR regions derived from rat mAb E specific for CPV into the corresponding CDR regions of feline pVITRO-Cat IgG-IgK.

### Recombinant protein expression

FreeStyle™ 293-F cells (Catalog number R79007, Thermo-Fisher Scientific, Waltham, MA) in suspension were maintained in FreeStyle™ 293 expression media in 125 ml Erlenmeyer flasks on an orbital shaker (135 rpm) in 8% CO_2_ at 37 °C according to the manufacturer’s instructions (Thermo-Fisher Scientific, Waltham, MA). Cells (30 ml at 1 × 10^6^ cells ml^−1^) in flasks were transiently transfected with antibody expression vectors (pVITRO-Cat IgG-IgL; pVITRO-Cat IgG-IgK; or pVITRO-felinized mAb E) using FreeStyle™ MAX transfection reagent (Thermo Fisher Scientific, Waltham, MA) according to the manufacturer’s instructions. Transfected cells were collected on day 7, centrifuged at 1,000 × *g* for 15 min, then the supernatants were filtered through 0.2 μm filters (Whatman, Shrewsbury, MA).

Recombinant antibodies were purified by loading Ig-containing supernatants onto a 1 ml HiTrap Protein A HP column. After washing in phosphate buffered saline (PBS), purified antibodies were eluted with 0.1 M glycine-HCl buffer (pH 2.7), and fractions were collected into tubes containing 1 M Tris-HCl (pH 9).

### SDS-polyacrylamide gel electrophoresis and immunoblot

The purity of the Cat IgG-IgL, Cat IgG-IgK and felinized mAb E was assessed by SDS-PAGE and immunoblot. One μg of antibody was electrophoresed on a 4–15% polyacrylamide gel under non-reducing conditions, and on a 4–20% gel under reducing conditions (10% β-mercaptoethanol). All samples in reducing or non-reducing sample buffer were heated for 5 min at 95 °C prior to electrophoresis. Proteins in gels were visualized with Coomassie brilliant blue staining.

For immunoblotting, SDS-PAGE gels were transferred to 0.45 μm PVDF membranes, blocked for 1 h with 5% milk in Tris-buffered saline with Tween 20 (TBST), and then membranes were probed with HRP-conjugated goat anti-cat IgG (H + L) (Invitrogen) or HRP-conjugated goat anti-rat IgG (H + L) overnight. After washing in TBST, bound HRP was detected with a chemiluminescent substrate (Thermo Fisher Scientific, Waltham, MA) and recorded with a ChemiDoc MP system (Bio-Rad, Hercules, CA).

### Hemagglutination inhibition (HAI) assay

To test the function of the engineered felinized mAb E, HAIs were used to determine the capacity of mAbs to inhibit CPV or feline panleukopenia virus (FPV) hemagglutination of feline red blood cells. Antibodies were serially diluted (12.5 μl of 80 μg ml^−1^ mAb E; felinized mAb E; or feline parental IgG-IgK) 1:1 in Bis-Tris buffered saline (BTBS; 0.1 mol Bis-Tris Base, 0.15 mol NaCl, 2.5 mmol MgCl_2_, 0.75 mmol CaCl_2_ and 0.1% BSA, pH = 5.87). Eight HA units of purified CPV or FPV empty capsids in 12.5 μl PBS were added to each well (25 μl total volume) in a 96-well V-bottom plate, and incubated for 1 h at room temperature. Feline red blood cells (0.5% in BTBS; 50 µl) were added to each well, and the plates were further incubated at 4 °C for 2 hours or overnight before being read.

### Bio-layer interferometry of mAb-CPV/FPV binding kinetics

To determine the antibody-antigen binding kinetics, we incubated purified CPV or FPV empty capsids with antibodies bound to a biosensor to examine the association and dissociation of virus-antibody complexes using a BLITz bio-layer interferometer (Pall ForteBio, Port Washington, NY). Briefly, Protein A biosensors (Pall ForteBio) were soaked in kinetics buffer (1 × PBS, 0.02% ovalbumin, 0.02% Tween 20) for 10 minutes. Biosensors were then inserted into the bio-layer interferometer and 1) washed in kinetics buffer for 30 s; 2) incubated with 4 μl of each antibody diluted in PBS for 300 s; 3) washed for 180 s in kinetics buffer; 4) incubated with 4 μl of 240 μg ml^−1^ of CPV or FPV empty capsids for 300 s; and 5) washed for 300 s in kinetics buffer. Because immunoglobulins from different species have different affinities for Protein A, we loaded different concentrations of antibodies (520 μg ml^−1^ of mAb E, 1.33 μg ml^−1^ cat IgG-IgK and 1.7 μg ml^−1^ of felinized mAb E) onto Protein A tips so that each antibody reached 1 nm of measured binding thickness after the 180 s wash in kinetics buffer. Achieving the same thickness of binding is a good indicator that the same amount of antibody is bound to the tip regardless of the different initial loading concentration. Subsequently, equal amounts of CPV and FPV were loaded onto tips to generate the binding kinetics curve. The experiments were repeated 3 times to obtain an averaged kinetics curve with standard error of the mean.

## Results

### Sequence analysis of full-length immunoglobulin heavy (IGH) chain mRNA transcripts

**Figure 1:**
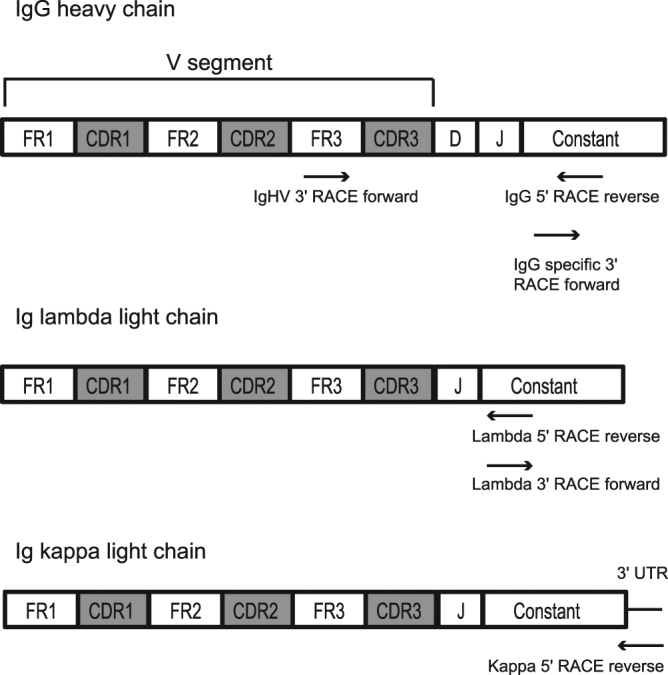
Schematic representation of the structure of feline Ig heavy and light chains. The positions of primers used to generate 5′ or 3′ RACE library are shown. FR, framework region; CDR, complementarity determining region.

**Figure 2:**
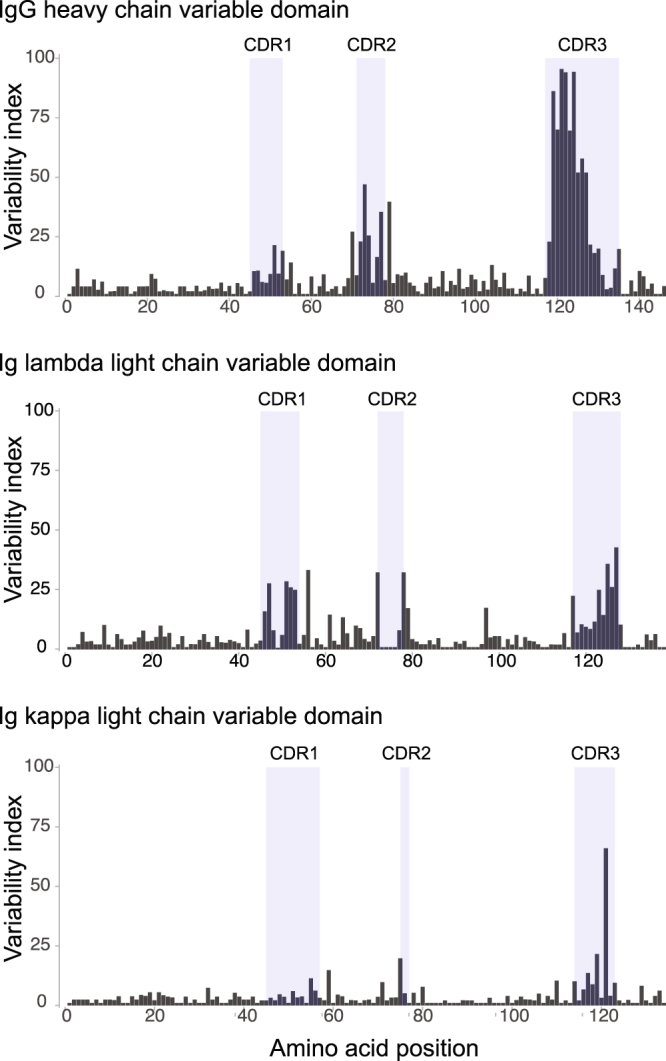
Variability plot of the variable domains of feline IgG heavy chain, Ig lambda and Ig kappa chain sequences. The hypervariable CDR 1–3 are shaded. The variability index was calculated by the number of different amino acids at a given residue divided by the frequency of the most common amino acid at that residue.

**Figure 3:**
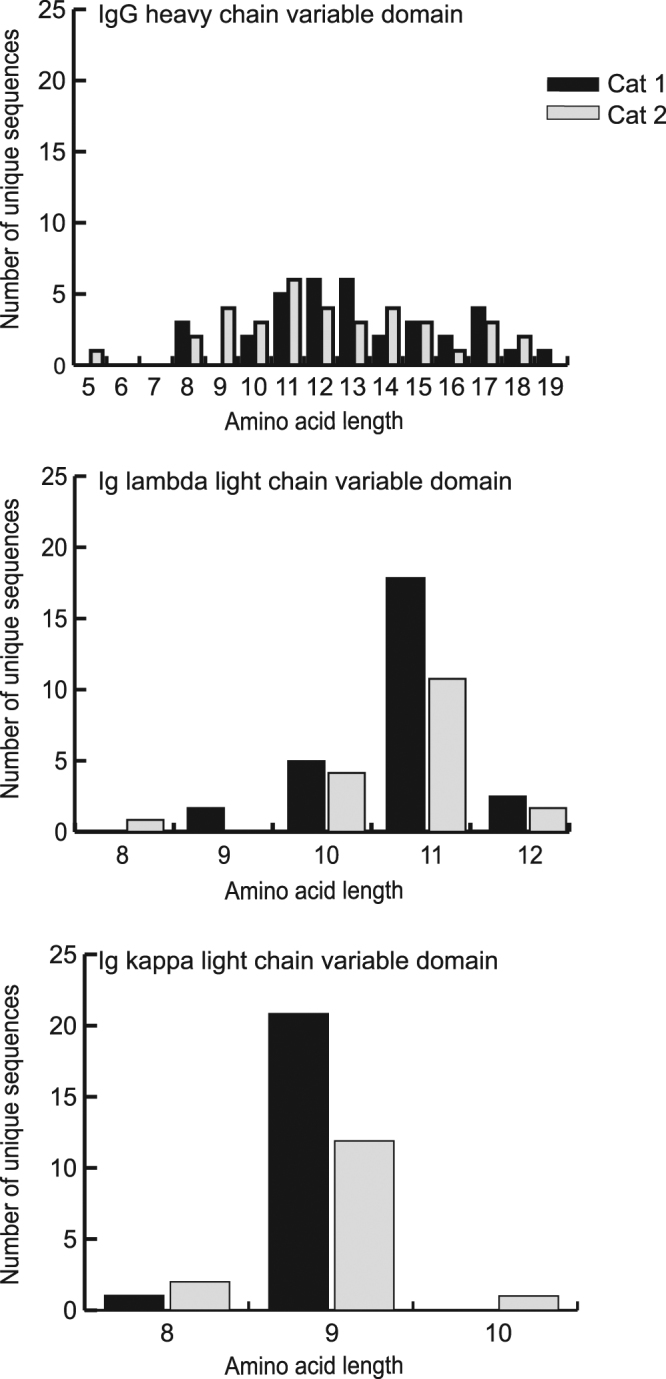
CDR3 length distribution of feline Ig sequences. The range of CDR3 amino acid length in IgG heavy chain, Ig lambda and kappa chain variable domains is shown and the number of unique sequences is plotted on the y-axis. Cat 1 clones are shown in black and cat 2 clones are shown in gray.

There are very few complete mRNA sequences available for feline IGH chains. Therefore, to identify full-length mRNA sequences of feline IGH chains, we extracted total RNA from PBMCs from two healthy cats and prepared 5′ and 3′ RACE libraries with primers designed using available cat Ig sequences (Fig. 1, Supplementary Table S1). Seventy-one unique IGH variable domain (IGHV) and 96 IGH constant domain (IGHC) RACE sequences were identified (Supplementary Table S2). Like other species, the highest sequence variability in the IGH was in the CDR3, with the most variable residues in CDR3 being closer to its amino terminus (Fig. 2). CDR1 and CDR2 were also more variable than the framework regions, but to a lesser degree than CDR3. CDR3 was generally longer (5–19 amino acids) than CDR1 and CDR2 (Supplementary Table S2 and Fig. 3). Seventy-five of the IGHC clones were of the isotype class IgG. Among these, one (1.3%) was subclass IgG2 (IGHG2) and the remainder (74 clones; 98.7%) were of subclass IgG1 (IGHG1). Although the Ig constant domain is considered “conserved”, we identified a few IGHG1a sequence variants within our clones (Supplementary Table S3).

The 71 IGHV sequences could be assigned to three IGHV subgroups (subgroup 1, 2 and 3; Supplementary Fig. S1) based on their sequence similarity, with the majority (72%) of the sequences belonging to the IGHV1 subgroup (Supplementary Table S4). The IGHV sequences assigned to each subgroup shared >75% nucleotide identity.

### Novel feline IgA heavy chain constant domain sequences

Twenty-one IGH RACE sequences corresponded to the IgA constant domain. IGHA sequences have not been previously reported for cats. The 21 IGHA constant domain sequences represented 22% of all the constant domain sequences (Supplementary Table S2). Eight IGHA amino acid variants were found, which are likely due to usage of different IGHA alleles and/or somatic mutations (Supplementary Table S5).

### Sequence analysis of mRNAs encoding lambda (IGL) and kappa (IGK) light chain immunoglobulins

No complete Ig lambda sequences were available when we began this project and only one kappa sequence was available. Therefore, we generated RACE libraries to recover these sequences. Different RACE libraries were used to identify the variable and constant domains of IGL sequences, but a single library was used to obtain full-length IGK sequences. As expected, the greatest variability in IGL was found in its CDR3 variable domain; and within the CDR3, residues towards the C-terminus were most variable (Fig. 2). Although variability in amino acids within the CDRs of IGL was easily discerned, we noted several residues that had high variability outside of the CDRs (e.g. residues 56, 61, 64 and 97) (Fig. 2). Variability of the IGK CDR3 was moderate-to-high, but CDR1 and CDR2 of IGK had low variability.

### Expressed IGH sequences map to three IGHC genes on chromosome B3 and nine IGHV genes on chromosomes B3 and D1

We mapped the sequences of Ig clones to the *Felis catus* reference genome (assembly Felis_catus_8.0) to determine the Ig germline gene usage and the extent of nucleotide diversity from the reference genome sequence. The sequences of Ig heavy chain constant domain clones of IGHG1a and IGHG2 mapped to feline genome chromosome B3 (NC_018728.2) (Supplementary Table S4). The expressed Ig heavy chain variable (IGHV) sequences mapped to 7 IGHV germline genes on chromosomes B3 (NC_018728.2) and 2 germline genes in D1 (NC_018732.2). Matches to chromosome B3 were expected based on previous mapping efforts^32^, but IGHV genes on chromosome D1 were novel (Supplementary Table S4) and may indicate an error in the genome assembly (see discussion). The IgA heavy chain (IGHA) constant domain sequences also mapped to feline genome chromosome B3 (NC_018728.2).

When we compared the IGHV clone sequences to the reference genome sequence, up to 10% nucleotide divergence was observed, although some sequences only differed from the reference genome sequence by one nucleotide (Supplementary Table S4). Sequences from 4 IGHV gene RACE clones had no significant matches in the feline reference genome.

### Expressed Ig lambda sequences mapped to thirty IGLV genes and five IGLC genes on chromosome D3 and other unmapped genomic scaffolds regions

The recovered Ig light chain (lambda and kappa) clone sequences were also mapped to the *Felis catus* 8.0 reference genome to identify gene usage. The usage of 30 Ig lambda chain variable (IGLV) genes were identified. Although some genes were only represented by one clone sequence, in some cases, the sequences derived from 6 clones were mapped to a single gene (Supplementary Table S6). Some clone sequences of the IGLV chain differed from the reference genome sequence by up to 5.5%, while others were identical to the reference genome sequence. We identified 5 subgroups among the 50 unique IGLV sequences based on each subgroup sharing more than 75% nucleotide sequence identity.

Prior to this work only one Ig lambda chain constant region (IGLC) sequence was available. We used this sequence to identify similar sequences in the feline reference genome, and all IGLC sequences obtained from the feline genome were used to design RACE primers. Because of this limitation, RACE primers were staggered in the IGLC gene, and thus the full-length IGLC chain was recovered by both comparison with the feline reference genome and by assembling the overlapping sequences obtained from RACE clones. These analyses revealed 5 IGLC genes on chromosome D3 (NC_018734) (Supplementary Table S7). While some IGLC sequences were identical to the feline genome, others varied by up to 4% nucleotide identity. All the identified IGLC sequences identified differed from the single pre-existing IGLC sequence.

### Expressed Ig kappa variable sequences map to six IGKV genes and one IGKC gene on chromosome A3

Although sequences for the Ig kappa (IGK) constant domain have been reported, the sequence diversity within the feline IGK variable domain has not been described. We obtained 37 unique sequences of full-length IGK transcripts. Comparison of the RACE clone sequences with the feline genome sequence identified the IGK locus on chromosome A3 (NC_018725.2). We detected 6 different IGKV germline genes that were used. The clone sequences varied by up to 7.4% in nucleotide identity from the feline reference germline sequence (Supplementary Table S8). Most (84%) IGKV RACE clone sequences belonged to IGKV subgroup 1, indicating that they shared >75% nucleotide identity. A second, less frequently (16%) expressed IGKV subgroup was detected in a single cat.

### Development of a vector to express recombinant feline Ig

**Figure 4:**
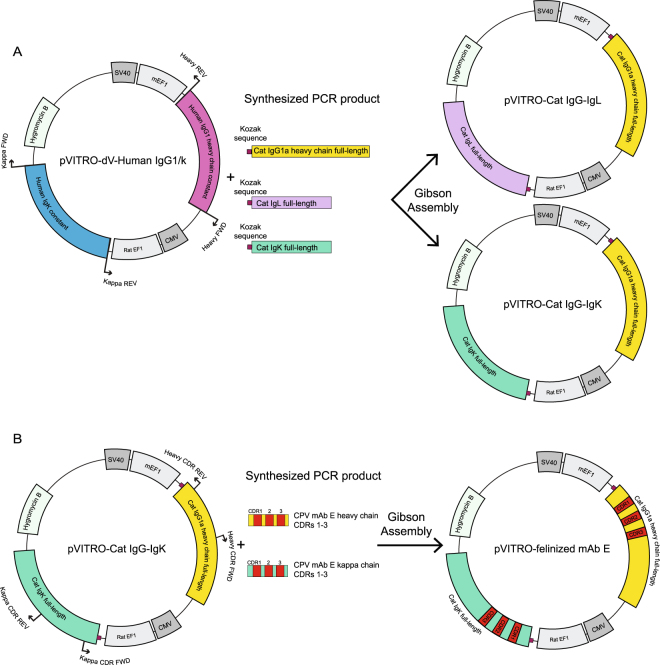
Schematic representation of the Gibson assembly cloning strategy for swapping antibody variable domains to generate feline expression vectors. (**A**) pVITRO-dV-Human IgG1/kappa vector was PCR linearized by flanking primer pairs. The synthetic PCR products of heavy, lambda or kappa chains were introduced into the vector using Gibson assembly. The resultant two plasmids pVITRO-Cat IgG-IgL and pVITRO-Cat IgG-IgK were used to express feline IgG-IgL and IgG-IgK antibodies, respectively. (**B**) Approach used to generate ‘felinized’ mAb E by swapping the CDR regions of mAb E into the feline Ig expression vector to generate pVITRO-felinized mAb E plasmid.

**Figure 5:**
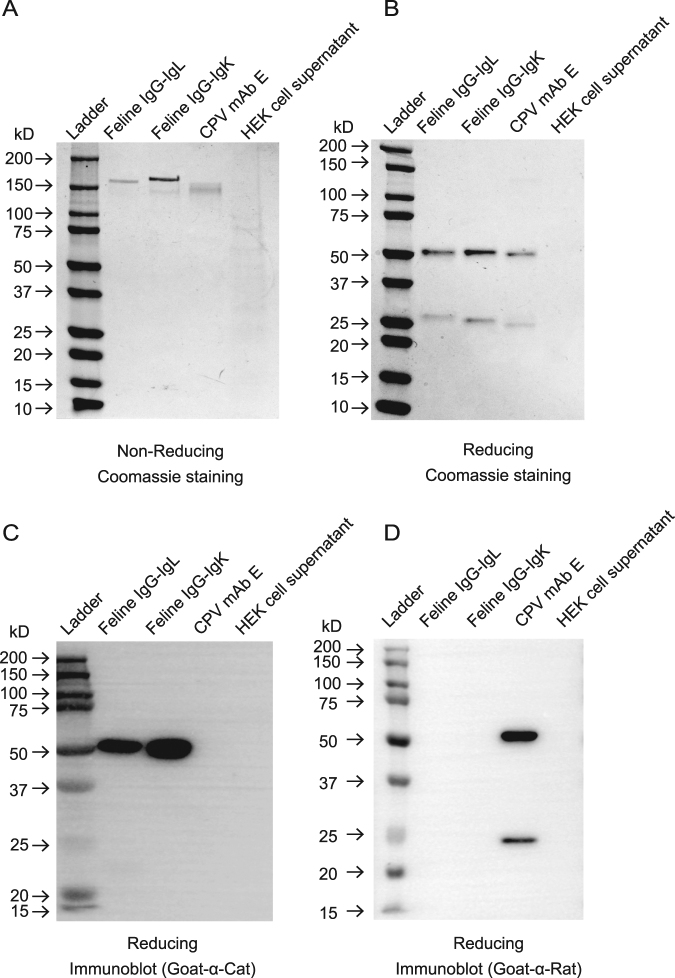
Recombinant feline antibodies assemble correctly and can be purified from transfected HEK cell supernatants. SDS-PAGE (**A**,**B**) and immunoblot (**C**,**D**) analysis of recombinant feline IgG-IgL and IgG-IgK under non-reducing (**A**) and reducing conditions (**B**–**D**). One µg of protein was loaded per lane. Proteins within gels (**A**,**B**) were visualized by Coomassie blue staining. PVDF membranes (**C**,**D**) were incubated with HRP-conjugated goat-anti-cat Ig or goat-anti-rat Ig antibodies, respectively. Feline IgL but not IgK chains were detected (not shown in this blot) (**C**). The antibody MWs are similar to monoclonal antibodies, indicating the secreted feline antibodies are properly folded and glycosylated.

One of our goals in identifying full-length feline heavy and light chain mRNA sequences was to test whether we could use these sequences to express and assemble a recombinant feline antibody. A bicistronic vector to express human Ig in mammalian tissue culture was developed by Dodev *et al*.^31^ and we adapted that system for feline antibody expression. Representative full-length sequences of feline IgG heavy chain and light chain (lambda, IgL and kappa, IgK) were cloned into the vector by Gibson assembly and the bicistronic vectors containing feline IgG-IgL or IgG-IgK were transfected into HEK cells (Fig. 4). The expression of feline IgG-IgL and IgG-IgK from the vectors was efficient yielding approximately 1 mg of Ig per 30 ml of transfected cells. Under non-reducing conditions, the molecular sizes of the expressed IgG/IgL and IgG/IgK were between 160–170 kDa (Fig. 5A). Because no free heavy or light chains were visible under non-reducing conditions, we concluded that the secreted antibody chains had successfully assembled into an intact antibody molecule. Under reducing conditions, heavy chains (~50 kDa) and light chains (~25 kDa) were visible (Fig. 5B). The feline origin of the secreted antibodies was confirmed by immunoblot using a goat anti-cat IgG (H + L) antibody (Fig. 5C). The heavy chain was easily detected, and the lambda light chain could be detected using a long exposure time (data not shown). However, we were unable to detect feline IgK light chain. We believe this is likely due to the specificity of the primary antibody used for detection. The purified feline Ig was not recognized by a goat anti-rat IgG (H + L) antibody (Fig. 5D). All unprocessed images are shown in Supplementary Fig. S2.

### Expression of a “felinized” monoclonal antibody specific for canine parvovirus and feline panleukopenia virus

**Figure 6:**
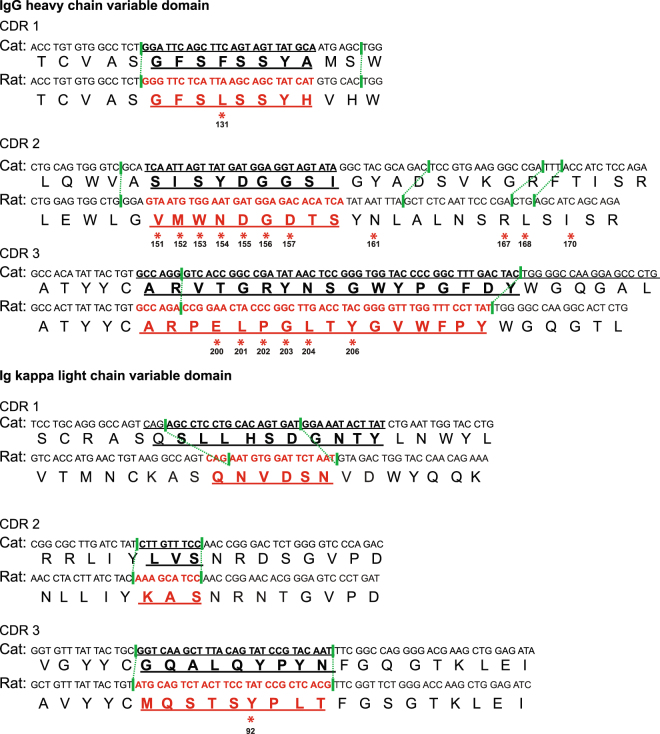
Amino acid and nucleotide sequences of the cat Ig and CPV mAb E heavy and light kappa chain were aligned and used to generate felinized mAb E. The CDRs determined by sequence analysis (Cat) or cryo-EM structural analysis (Rat) are emboldened in black and red, respectively. The contact residues of mAb E to CPV capsid are indicated with asterisks and numbered according to the mAb Ig amino acid sequences. The green lines show the swapped CDR regions.

To test the functional utility of the expression vector, we designed a felinized IgG in which the CDRs of the feline IgG heavy and light kappa chains were replaced with the CDRs from a well-characterized rat mAb specific for canine parvovirus and feline panleukopenia virus (mAb E)^25^. We used sequence alignment and a cryo-EM structure of a complex of mAb E with canine parvovirus^33^ to guide our choices of residues within the CDR and flanking framework regions to swap. Our initial attempt at CDR swapping included 11 amino acids of framework region flanking the heavy chain CDR2 that contacted the CPV capsid. However, the inclusion of these framework residues made the expressed feline Ig unstable and the resultant protein misfolded (data not shown). We therefore limited the numbers of residues swapped in the framework region. The mAb E heavy CDRs1–3 (301 nt) and kappa CDRs 1–3 (265 nt) were synthesized as PCR products and swapped with corresponding sequences of the vector pVITRO-Cat IgG-Cat IgK using Gibson cloning (Fig. 6).

**Figure 7:**
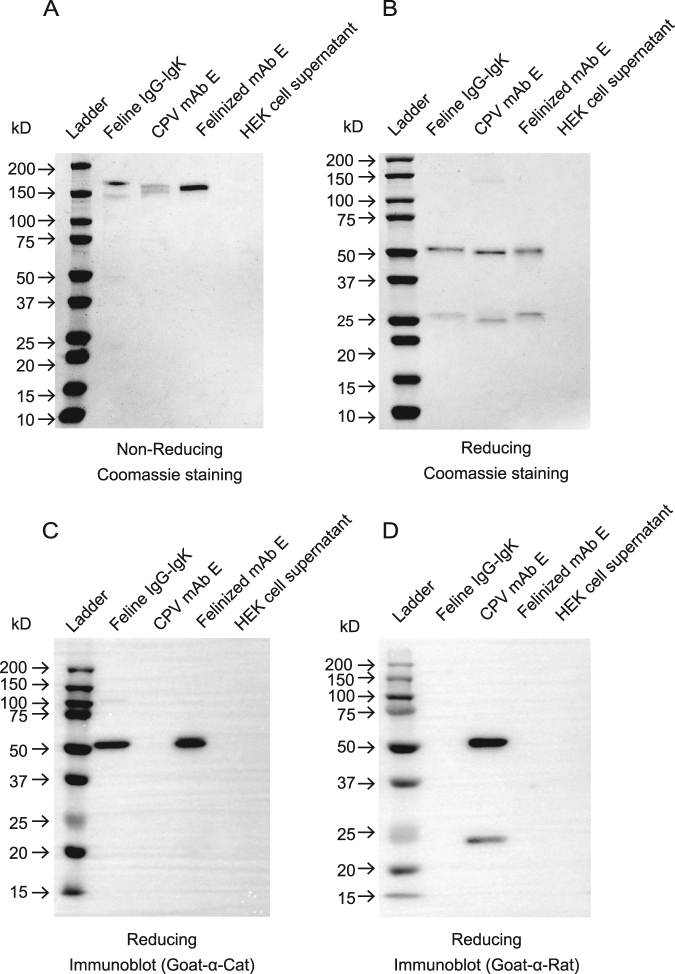
Purified felinized mAb E is expressed and assembles correctly. SDS-PAGE (**A**,**B**) and Immunoblot (**C**,**D**) analysis of felinized mAb E under non-reducing (**A**) and reducing conditions (**B**–**D**). One µg of protein was loaded per lane. Proteins in gels (**A**,**B**) were visualized by Coomassie blue staining. PVDF membranes (**C**,**D**) were incubated with HRP-conjugated goat anti-cat Ig or goat anti-rat Ig antibodies, respectively.

When expressed under the same conditions as the wild type cat antibody, the felinized mAb E yield was substantially lower, with approximately 20 μg of Ig yield per 30 ml of transfected cells. Under non-reducing conditions, the felinized antibody ran as a single 150 kDa band on SDS-PAGE (Fig. 7A). The 52 kDa heavy chain and 25 kDa light chain were visible on stained SDS-PAGE gels under reducing conditions (Fig. 7B). Like the unaltered recombinant feline Ig, the heavy chain of the felinized antibody but not the light IgK chain was detected using goat-anti cat IgG (H + L) antibody by immunoblotting (Fig. 7C). The felinized antibody was not detected with goat anti-rat IgG (H + L) antibody (Fig. 7D). All unprocessed images are shown in Supplementary Fig. S3.

**Figure 8:**
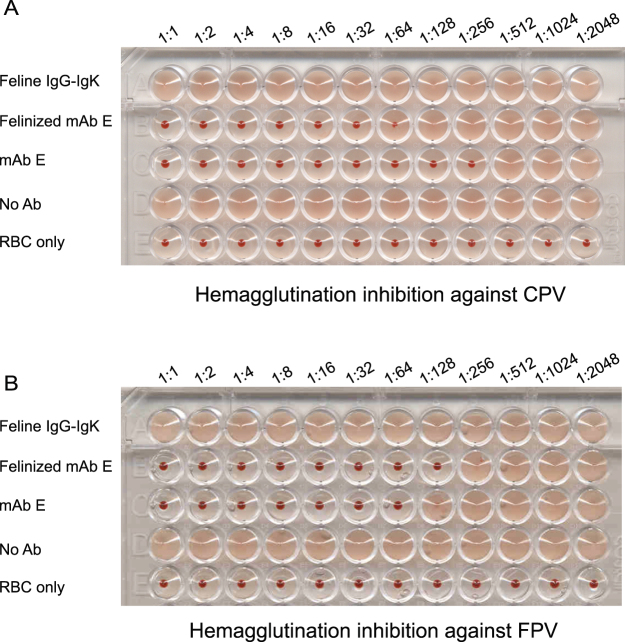
Felinized mAb E inhibits hemagglutination by (**A**) CPV and (**B**) FPV. Eight HA units of purified CPV or FPV empty capsids were added to two-fold serial diluted antibodies starting at 80 µg/ml. After incubation at room temperature for 1 hr, 50 µl of 0.5% feline red blood cells were added to each well and the plates were incubated at 4 °C overnight. Experiments were done three times independently. A representative plate is shown.

Having successfully expressed the felinized mAb E in tissue culture, we further tested the capacity of this antibody to recognize CPV and FPV using a hemagglutination inhibition assay (HAI). Monoclonal antibody E prevents hemagglutination of feline RBCs by CPV or FPV empty capsids. As expected, the wild-type cat IgG-IgK did not inhibit CPV or FPV hemagglutination. In contrast, the felinized mAb E inhibited hemagglutination by CPV or FPV. The HAI titer of the felinized mAb E for CPV was lower than mAb E, but was higher than mAb E for FPV (Fig. 8).

**Figure 9:**
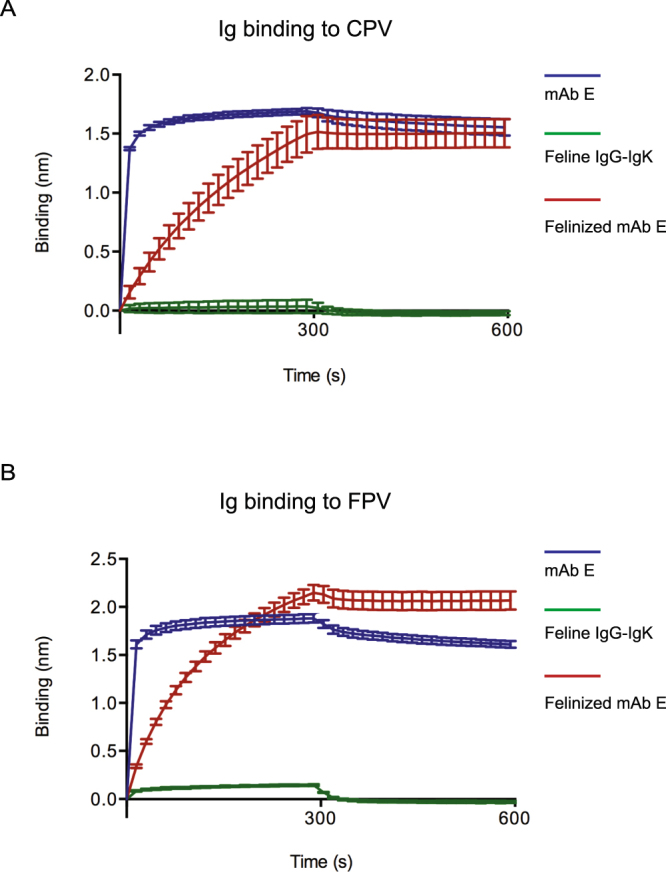
Binding of (**A**) CPV or (**B**) FPV empty capsid to antibodies using biolayer interferometry. The sensorgram data show the association of CPV or FPV with mAb E, feline IgG-IgK or felinized mAb E for 300 sec of incubation, followed by dissociation for 300 sec. The curves and error bars represent the averaged data from 3 independent experiments. The error bars display the standard error of the mean.

We further examined the binding kinetics of the Ig to CPV and FPV using biolayer interferometry, an *in vitro* assay that shows the rates at which IgG bound to a biosensor’s surface associates and disassociates with viral capsids in solution. Wild-type mAb E bound rapidly to CPV and FPV. In contrast, the felinized mAb E bound more slowly, but reached similar levels of overall binding after the 300 seconds association phase (Fig. 9). Disassociation curves for both wild-type mAb E and felinized mAb E were relatively flat indicating the antibodies remained bound. Of note, the overall amount of FPV bound to felinized mAb E was greater than the parental mAb E, as shown by higher binding levels after the end of the disassociation phase (Fig. 9B). The swapped CDR may change the shape of the Ig slightly and result in this effect. As expected, the parental feline IgG did not bind to CPV and FPV.

## Discussion

Engineered human mAbs are a rapidly growing category of therapeutic agents being used for the treatment of cancer, immunological disorders and infectious diseases^34^. As of October 2016, there were 65 therapeutic mAbs approved by the FDA for human therapy^35^. The therapeutic use of mAbs in veterinary medicine is still limited but of growing interest. Candidate antibodies for treatment of canine B-cell lymphoma and “caninized” anti-nerve growth factor (NGF) have been reported in veterinary medicine. The anti-NGF antibody has been evaluated for its utility in clinical studies of osteoarthritic dogs^36–38^. So far, only one felinized mAb (anti-NGF) has been reported, and *in vivo* studies have shown that the antibody could provide safe analgesia in cats^39^. To prepare that antibody, two separate expression vectors were used to express Ig heavy and light chains. The goal of our study was to develop a generalized feline expression vector that can be used to engineer felinized mAbs. The vector we have adapted could be used to insert CDRs from well characterized human or murine antibodies into a feline Ig backbone. Such antibodies are likely to prove useful in a variety of therapeutic applications, including cancer therapy, treatment of infectious diseases and other conditions.

We successfully identified novel full-length IGHA, IGLV, IGLC, and IGK sequences. Although the Ig sequences we obtained from 2 cats expand our knowledge of the feline Ig repertoire and Ig gene usage, more extensive studies will be needed using different cat breeds to determine if the data we have collected can be broadly applied. We observed expression from 3 IGHV subgroups, with the majority (72%) of these sequences belonging to the IGHV1 subgroup. These findings indicates that feline IGHV diversity is derived from the use of more germline subgroups genes than the canine IGHV repertoire, in which over 90% of the sequences are derived from a single canine IGHV subgroup (IGHV1), and the remainder from IGHV2^40^.

The nine IGHV genes used were located on chromosomes B3 (NC_018728.2) and D1 (NC_018732.2). Usage of IGHV genes on B3 was expected, but usage of genes on chromosome D1 was unexpected, as IGHV genes were not expected to be present in more than one chromosome. We speculate that the variable gene mapping to chromosome D1 is due to a genome sequence assembly error, since no Ig heavy chain constant domains mapped to chromosome D1, and recombination between V, D, and J-C gene segments is unlikely to occur with transcripts derived from two chromosomes. Mapping immunoglobulin genes is challenging when assembling a genome because of their tandem array and conserved sequences. The bovine genome has 2 IGH loci on different chromosomes. A functional IGH locus is present on bovine chromosome BTA21. The second locus on bovine chromosome BTA11 is truncated^41^. Thus, a second functional feline IGH locus on another chromosome seems unlikely. The full-length feline IgM and IgA heavy chain constant domain genes were found on chromosome B3. The full-length IGHG constant domain also mapped to B3 although fragments (≤63% of the lGHG constant domain length) mapped to a yet unlocalized genome scaffold sequence. We also identified four clone sequences that had no significant hits within the feline reference genome; it is possible that the individual cat used to generate the reference genome lacked these genes; alternatively, gaps in the feline reference genome assembly may explain the absence of these sequences.

The dominance of IGHG1 usage (≥98% expressed IGHG transcripts) identified by us and others^17,42^ in feline peripheral blood cells is in striking contrast to the number of IGHG isotypes expressed by other species, generally ranging from 4 to 8 in cattle, mouse, human, pig, horse, and elephant^43–47^. This indicates that Fc receptor interactions and effector functions of IgG proteins (complement fixation, opsonization, triggering of antibody dependent cellular cytotoxicity) must all be determined by a single feline IGHG1 constant domain, contrasting with other species in which different isotypes exert distinct effector functions^48^. Only one feline IGHA constant domain gene was identified from this work, although different alleles may exist. The genome of some species harbor multiple IGHA constant domain genes, such as the 13 different IGHA constant domain genes found in rabbits^49^.

One of the most intriguing observations was the large number of variable genes used to encode the lambda light chain variable region. Thirty IGLV genes were mapped suggesting that the IGL variability in cats is largely dependent on the number of genes used rather than the variation at each individual amino acid position. It has been posited that the choice of Ig light chain used is a dominant phenotype in a species and may be associated with the complexity of the Ig light chain loci^50^. This hypothesis is based on observations in mice and humans, where dominant light chain protein expression corresponds to the light chain with the highest number of functional variable germline genes (i.e., in mice >95% of Ig light chains are kappa and the mouse genome contains at least 67 functional IGKV genes and only 3 IGLV genes; in humans ~60% of Ig light chains are kappa, and the human genome encodes 40 functional IGKV genes and 30 IGLV genes)^50^. This appears to hold true for the feline Ig light chain repertoire, as lambda expression dominated over kappa with more IGLV genes expressed than IGKV genes (5 IGLV subgroups expressed from 30 genes versus 2 IGKV subgroups from 6 genes). It should be noted, however, that comprehensive genomic IGLV and IGKV mapping has not yet been performed.

We found that the yield of felinized mAb E was much lower than the feline IgG-IgK under similar transfection conditions. It is possible that the modification of Ig sequences interfered with the efficient final assembly of functional Ig molecules. When swapping CDRs, it is important to maintain the balance between the framework integrity and the numbers of contact residues within the framework region that are changed. Balancing framework integrity with the loss of some contact residues is an accepted practice for engineering chimeric Ig molecules.

Antibody-antigen recognition is a dynamic process and can be of “lock-and-key”^51^ or of “induced fit” type^52^. Our binding kinetics data showed that the felinized mAb E had a much slower association to CPV or FPV than mAb E. This observation suggests that the initial binding of felinized mAb E with CPV or FPV may require conformational changes of the antibody-capsid complex, which subsequently facilitates additional antibody binding. Another possibility is that the felinized antibody has a different structure than the wild type rat antibody, and therefore it binds differently. Although there was a difference between association of mAb E and felinized mAb E, there was no difference in the functional capacity of these two antibodies to inhibit CPV/FPV hemagglutination. The functional capability of the two antibodies is consistent with studies showing that the off-rate inversely correlates with neutralization titer after vaccination^53^. A series of CDR-mutations in a mouse-human chimeric antibody showed that neutralization and signaling efficiency dropped proportionally as the off-rate increased^54^. Both mAb E and felinized mAb had similar dissociation curves, which may explain their equivalent capacities to inhibit CPV/FPV hemagglutination.

In summary, we have characterized feline Ig sequences and mapped their gene usage. Feline Ig was expressed using a bicistronic expression vector. As a proof of principle, we generated a felinized mAb specific for CPV and FPV by swapping the CDRs from a rat anti-CPV mAb into the expression vector backbone. The felinized mAb was fully functional and maintained its antigen-recognition capacity. Such a feline expression vector will facilitate development of innovative antibody-based therapeutics in cats.

## Electronic supplementary material

Supplementary Information
