# Supplementary material for: Sequence analysis of feline immunoglobulin mRNAs and the development of a felinized monoclonal antibody specific to feline panleukopenia virus
Source: Sci Rep. 2017 Oct 5;7:12713. doi: 10.1038/s41598-017-12725-5 (PMC5629197; doi:10.1038/s41598-017-12725-5)
Supplement: Supplementary file 1 — Supplementary Information [file 41598_2017_12725_MOESM1_ESM.pdf]

Sequence analysis of feline immunoglobulin mRNAs and the development of a felinized monoclonal antibody specific to feline panleukopenia virus

Zhengchun Lu,[1†] Rebecca L. Tallmadge,[2†] Heather M. Callaway[1], M. Julia B. Felippe,[2] and John SL. Parker[1*]

Baker Institute for Animal Health[1] and Department of Clinical Sciences,[2] College of Veterinary Medicine, Cornell University, Ithaca, NY 14853, USA

Supplementary Table S1. Primers used in RACE library

Primer name	5′-3′ sequences	Target region
IgG 5′ RACE reverse	CACCGGCTCAGGGAAGTAGCCTAAC	IGHC
IgHV 3′ RACE forward	GAGGACACGGCCRYATATTACTGT	FR3 region of IGHV
IgG specific 3′ RACE forward	AMGGCCYCATCGGTGTTCCCACK	IGHC
Lambda 5′ RACE reverse	GTAGCTGCTGGCCGCGTACTTGTTGT	IGLC
Lambda 3′ RACE forward	GTGYCTCATCAGTGACTTCTACCC	IGLC
Kappa 5′ RACE reverse	GAGGGAGACAGAGAGTCTGGGACTG	3′ UTR of IGKC

Supplementary Table S2. Summary of feline immunoglobulin sequences determined by RACE clone sequencing

	Cat 1	Cat 2
**Donor information**		
Age	10-month-old	6-month-old
Sex	Male	Female
Breed	Domestic short hair	Domestic short hair
**Immunoglobulin heavy chain (IGH)**		
**Variable domain**		
No. of unique sequences	35	36
Median length of coding sequence (nt)*	345	345
CDR1 length (amino acid)	8-9	8-9
CDR2 length (amino acid)	5-8	5-8
CDR3 length (amino acid)	8-19	5-18
**Constant domain**		
No. of unique IgG sequences	37	38
No. of IgG1a sequences	36	38
No. of IgG2 sequences	1	0
Median length of IgG coding sequence (nt)**	1,008	1,008
No. of unique IgA sequences	9	12
Median length of IgA coding sequence (nt)	1,032	1,032
**Immunoglobulin lambda light chain (IGL)**		
**Variable domain**		
No. of unique sequences	29	21
Median length of coding sequence (nt)*	309	318
CDR1 length (amino acid)	3-5	3-5

CDR2 length (amino acid)	3-7	3-7
CDR3 length (amino acid)	9-12	8-12
**Constant domain**		
No. of unique sequences	19	16
Median length of coding sequence (nt)***	320	320
**Immunoglobulin kappa light chain (IGK)**		
No. of unique sequences	22	15
Median length of kappa chain full-length coding sequence (nt)	729	729
CDR1 length (amino acid)	6-11	6-11
CDR2 length (amino acid)	3	3
CDR3 length (amino acid)	8-9	8-10

* Median length of variable domain coding sequence is from the start codon to the 3′ end of framework 3
** Median IgG heavy chain constant domain coding sequence is determined from RACE clones that contained the full-length constant domain
*** Median lambda constant domain coding sequence is determined from the full-length constant domain, obtained from assembly of 5′ and 3′ RACE clone sequences

Supplementary Table S3. Amino acid variants in IgG constant domain sequences

	**IgG constant domain amino acid position**												
	**3**	**4**	**11**	**18**	**26**	**200**	**243**	**257**	**259**	**297**	**300**	**304**	**307**
Clone sequence consensus*	T	T	L	T	A	L	E	S	H	K	V	H	R
cat 1-IGHV-18	**P**	**K**	**R**	**I**	**I**	-	-	-	-	-	-	-	-
cat 1-IGHG-23	-	-	-	•	•	**V**	•	•	•	•	•	•	•
cat 2-IGHG-23	-	-	-	•	•	•	**K**	•	•	•	•	•	•
cat 2-IGHG-1	•	•	•	•	•	•	•	**G**	**Y**	**R**	**M**	**R**	**S**

* The consensus sequence provided in the table was derived from alignment of all IGHG RACE clone sequences. Dash '-' indicates sequence unknown due to RACE primer position. Dot "•" indicates the same amino acid as consensus sequence. Variant amino acids are highlighted as bold.

Supplementary Table S4. Genome mapping and usage of Ig heavy chain variable and constant domain (IGHV and IGHC) genes obtained from RACE clone sequencing

Ig region	Genome location			All sequences		Cat 1		Cat 2	
	Chromosome (GenBank accession)	Start position	End position	No. of sequences (%)	Germline identity (%)*	No. of sequences	IGHV subgroup**	No. of sequences	IGHV subgroup**
**Heavy chain variable (IGHV)**	B3 (NC_018728.2)	147,301,185	147,300,734	16 (22%)	92-100%	6	2	10	2
	B3 (NC_018728.2)	147,334,202	147,333,685	3 (4%)	95-98%	0	NA	3	1
	B3 (NC_018728.2)	147,463,145	147,462,631	6 (8%)	91-94%	4	1	2	1
	B3 (NC_018728.2)	147,480,663	147,480,146	5 (7%)	91-95%	3	1	2	1
	B3 (NC_018728.2)	147,503,206	147,502,683	1 (1%)	92%	1	1	0	NA
	B3 (NC_018728.2)	147,528,950	147,528,433	1 (1%)	97%	1	1	0	NA
	B3 (NC_018728.2)	147,692,816	147,692,293	3 (4%)	94-99%	2	1	1	1
	D1 (NC_018732.2)	22,921,465	22,921,987	25 (35%)	90-97%	13	1	12	1
	D1 (NC_018732.2)	22,934,469	22,934,989	7 (10%)	90-94%	3	1	4	1
	No significant hit in reference genome			4 (6%)	NA	2	3	2	3
**Heavy chain constant (IGHC)**									
IGHG1a	B3 (NC_018728.2)	147,144,575	147,146,215	74	99-100%	36	NA	38	NA
IGHG2	B3 (NC_018728.2)	147,179,783	147,181,383	1	100%	1	NA	0	NA
IGHA	B3 (NC_018728.2)	147,126,383	147,128,025	21	99-100%	9	NA	12	NA

* Range of percentage of germline identity was determined by comparison of nucleotide sequences to Felis catus reference genome (assembly Felis_catus_8.0)
** Variable gene subgroups were comprised of Ig heavy chain variable domain sequences sharing >75% nucleotide identity
'NA' indicates not applicable

Supplementary Table S5. Amino acid variants in IgA constant domain sequences

	IgA constant domain amino acid position							
	**22**	**65**	**69**	**87**	**107**	**156**	**187**	**234**
Clone sequence consensus	V	T	L	E	Q	R	R	E
cat 1-IGHA-3, 4, 5, 8<br>cat 2-IGHA-6, 7, 8, 11	**A**	•	•	•	•	•	•	•
cat 1-IGHA-3, 4, 5, 8<br>cat 2-IGHA-2, 6, 7, 8, 11	•	**M**	•	•	•	•	•	•
cat 2-IGHA-11	•	•	**P**	•	•	•	•	•
cat 2-IGHA-1, 3, 4, 9	•	•	•	**Q**	•	•	•	•
cat 1-IGHA-4, 5, 8<br>cat 2-IGHA-2, 6, 7, 8, 11, 12	•	•	•	•	**P**	•	•	•
cat 1-IGHA-4, 5, 8<br>cat 2-IGHA-2, 6, 7, 8, 12	•	•	•	•	•	**K**	•	•
cat 1-IGHA-6	•	•	•	•	•	•	**H**	•
cat 2-IGHA-12	•	•	•	•	•	•	•	**G**

Dot "•" indicates the same amino acid as consensus sequence. Variant amino acids are highlighted as bold.

Supplementary Table S6. Genome mapping and usage of Ig lambda chain variable domain (IGLV) genes obtained from RACE clone sequencing

Genome location			All sequences		Cat 1		Cat 2	
**Chromosome (GenBank accession)**	**Start position**	**End position**	**No. of sequences (%)**	**Germline identity (%)***	**No. of sequences**	**IGLV subgroup****	**No. of sequences**	**IGLV subgroup****
D3 (NC_018734)	20,177,819	20,177,197	1 (2%)	100%	0	NA	1	2
D3 (NC_018734)	20,232,586	20,231,887	2 (4%)	96-97%	1	2	1	2
D3 (NC_018734)	20,248,033	20,247,283	1 (2%)	97%	0	NA	1	2
D3 (NC_018734)	20,258,353	20,257,610	2 (4%)	96-97%	1	2	1	2
D3 (NC_018734)	20,294,947	20,294,440	1 (2%)	100%	1	4	0	NA
D3 (NC_018734)	20,405,447	20,404,696	1 (2%)	94%	0	NA	1	2
D3 (NC_018734)	20,447,506	20,447,027	1 (2%)	99%	0	NA	1	5
D3 (NC_018734)	20,610,242	20,609,759	3 (6%)	95-98%	3	1	0	NA
D3 (NC_018734)	20,635,351	20,634,856	3 (6%)	98-100%	1	1	2	1
D3 (NC_018734)	20,706,336	20,705,868	2 (4%)	98-99%	1	1	1	1
D3 (NC_018734)	20,714,210	20,713,765	1 (2%)	96%	0	NA	1	1
D3 (NC_018734)	20,721,897	20,721,424	1 (2%)	96%	0	NA	1	1
D3 (NC_018734)	20,723,803	20,723,352	2 (4%)	97-98%	1	1	1	1
D3 (NC_018734)	20,802,815	20,802,344	2 (4%)	98-100%	0	NA	2	1
D3 (NC_018734)	20,802,813	20,802,344	1 (2%)	98%	1	1	0	NA
D3 (NC_018734)	20,808,197	20,807,731	1 (2%)	97%	1	1	0	NA
D3 (NC_018734)	20,840,083	20,839,623	1 (2%)	99%	1	3	0	NA
D3 (NC_018734)	20,845,910	20,845,432	2 (4%)	98-99%	1	1	1	1
D3 (NC_018734)	20,893,791	20,893,295	2 (4%)	95-98%	1	3	1	3
Unplaced scaffold (NT_318490)	5,956	6,425	1 (2%)	98%	1	1	0	NA

Unplaced scaffold (NT_318490)	13,034	13,499	1 (2%)	96%	1	1	0	NA
Unplaced scaffold (NT_318509)	2,427	1,957	1 (2%)	98%	0	NA	1	1
Unplaced scaffold (NT_360730)	2,428	2,920	2 (4%)	95-98%	1	1	1	1
Unplaced scaffold (NT_363715)	68	540	4 (8%)	96-99%	2	1	2	1
Unplaced scaffold (NT_363889)	469	939	1 (2%)	97%	0	NA	1	1
Unplaced scaffold (NT_383395)	not in scaffold	451	1 (2%)	99%	1	1	0	NA
Unplaced scaffold (NT_384526)	not in scaffold	412	1 (2%)	98%	1	1	0	NA
Unplaced scaffold (NT_390420)	687	216	6 (12%)	97-100%	6	1	0	NA
Unplaced scaffold (NT_411266)	not in scaffold	544	1 (2%)	97%	1	2	0	NA
Unplaced scaffold (NT_440987)	not in scaffold	326	1 (2%)	99%	1	1	0	NA

* Range of percentage of germline identity was determined by comparison of nucleotide sequences to Felis catus reference genome (assembly Felis_catus_8.0)
** Variable gene subgroups were comprised of sequences sharing >75% nucleotide identity
'NA' indicates not applicable

Supplementary Table S7. Genome mapping and usage of Ig lambda chain constant domain (IGLC) genes obtained from RACE clone sequencing

Chromosome (GenBank accession)	Genome location				All sequences		Cat 1		Cat 2	
	5′ RACE sequences		3′ RACE sequences		No. of sequences 5′ (%) + 3′(%)	Germline identity (%)*	No. of 5′ sequences	No. of 3′ sequences	No. of 5′ sequences	No. of 3′ sequences
	Start position	End position	Start position	End position						
D3 (NC_018734)	20,128,821	20,128,636	20,128,718	20,128,338	7 (33%) + 5 (14%)	96-100%	2	2	5	3
D3 (NC_018734)	20,137,345	20,137,160	20,137,266	20,136,860	2 (10%) + 5 (14%)	98-100%	1	2	1	3
D3 (NC_018734)	20,142,222	20,142,037	20,142,144	20,141,748	6 (29%) + 2 (6%)	96-100%	1	2	5	0
D3 (NC_018734)	20,150,763	20,150,578	20,150,685	20,150,276	2 (10%) + 9 (26%)	98-100%	1	5	1	4
D3 (NC_018734)	20,155,061	20,154,876	20,154,983	20,154,607	4 (19%) + 14(40%)	97-100%	3	8	1	6

* Range of percentage of germline identity was determined by comparison of nucleotide sequences to Felis catus reference genome (assembly Felis_catus_8.0)

Supplementary Table S8. Genome mapping and usage of Ig kappa chain (IGKV and IGKC) genes obtained from RACE clone sequencing

Genome location			All sequences		Cat 1		Cat 2	
**Chromosome (GenBank accession)**	**Start position**	**End position**	**No. of sequences (%)**	**Germline identity (%)***	**No. of sequences**	**IGKV subgroup****	**No. of sequences**	**IGKV subgroup****
**Kappa chain variable (IGKV)**								
A3 (NC_018725.2)	102,179,663	102,180,215	6 (16%)	94-100%	6	2	0	NA
A3 (NC_018725.2)	102,202,884	102,202,153	14 (38%)	93-99%	8	1	6	1
A3 (NC_018725.2)	102,216,748	102,216,017	11 (30%)	93-100%	4	1	7	1
A3 (NC_018725.2)	102,268,844	102,268,049	2 (5%)	98-99%	2	1	0	NA
A3 (NC_018725.2)	114,967,828	114,967,071	1 (3%)	98%	0	NA	1	1
A3 (NC_018725.2)	114,982,692	114,981,976	3 (8%)	99-100%	2	1	1	1
**Kappa chain constant (IGKC)**								
A3 (NC_018725.2)	102,159,103	102,159,458	37 (100%)	100%	22	NA	15	NA

* Range of percent germline identity determined by comparison to Felis catus reference genome (assembly Felis_catus_8.0)
** Variable gene subgroups were comprised of sequences sharing >75% nucleotide identity
'NA' indicates not applicable

Supplementary Figure S1

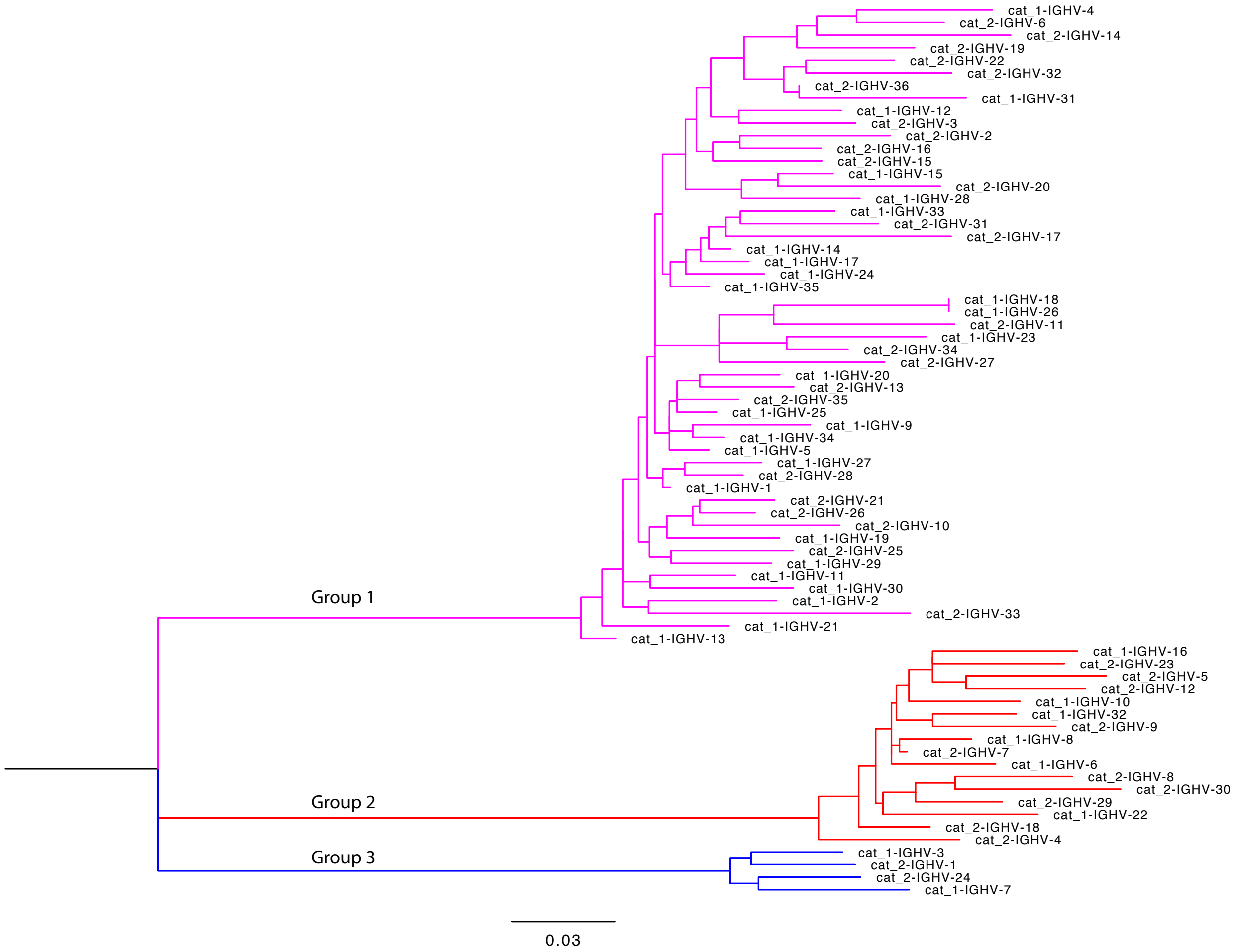

Phylogenetic analysis of 71 clone sequences of IGHV obtained from 2 cats using the Maximum Likelihood method on the Tamura-Nei model. Sequence analysis was conducted in MEGA7[30] and illustrated with FigTree (version 1.4.3). The tree is drawn to scale, with branch lengths measured in the number of substitutions per site. Three subgroups are colored magenta, red and blue.

Supplementary Figure S2

Unprocessed Fig 5A

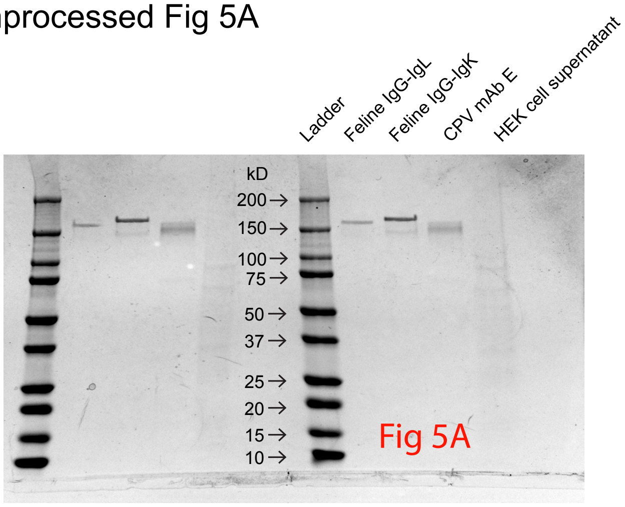

Full-length SDS-PAGE gel of Fig 5A as dectected by Coomassie staining autoexposure

Unprocessed Fig 5B

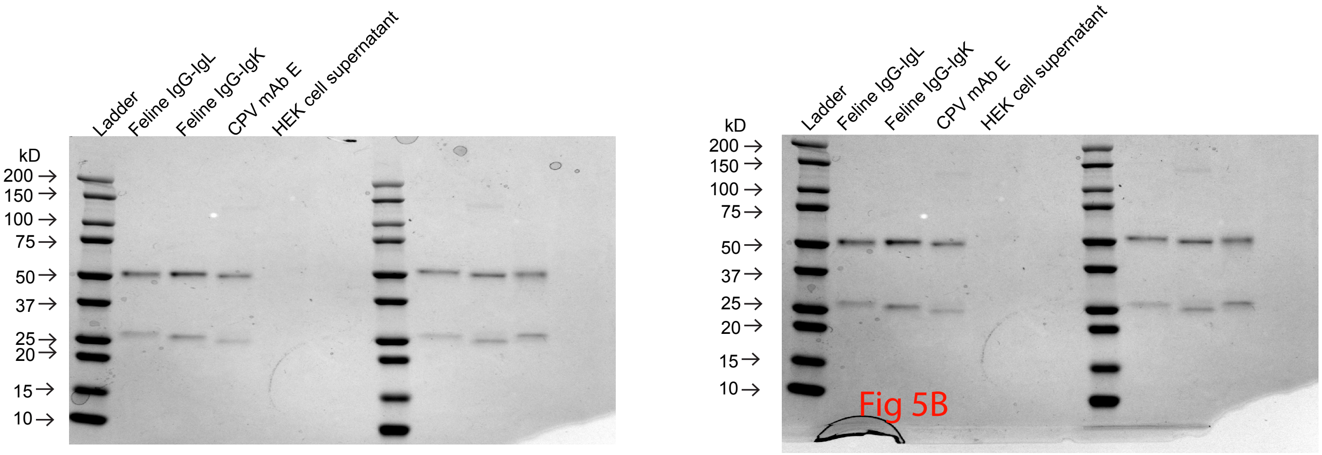

Full-length SDS-PAGE gel of Fig 5B as dectected by Coomassie staining autoexposure. The left gel was autoexposed to detect faint bands. The right gel was autoexposed to detect intense bands. The right left pannel was cropped to generate Fig 5B.

Unprocessed Fig 5C

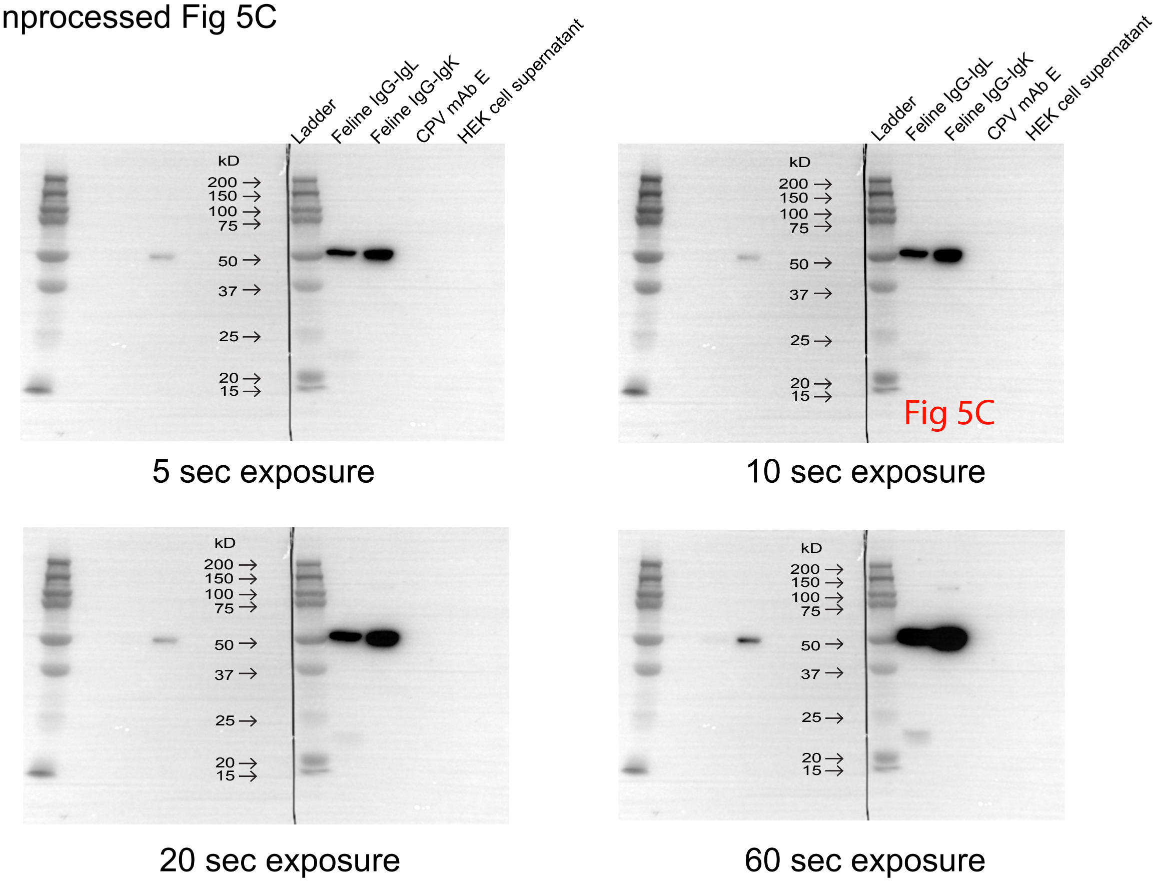

Full-length immunoblot of Fig 5C as dectected by goat-a-cat-HRP conjugated antibody. Blots with different exposure time were shown. The right panel in the blot with 10 sec exposure was cropped to generate Fig 5C.

Unprocessed Fig 5D

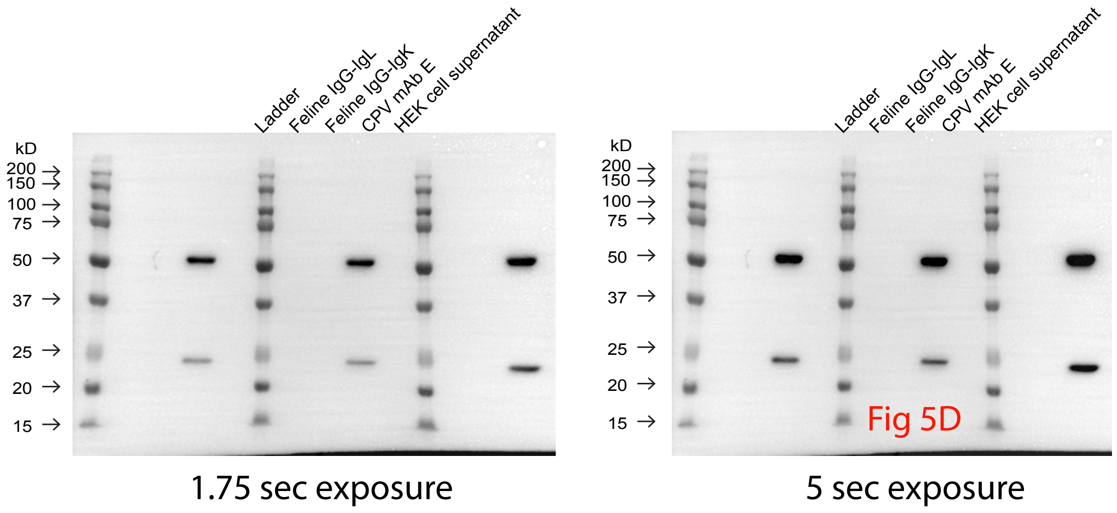

Full-length immunoblot of Fig 5D as dectected by goat-a-rat-HRP conjugated antibody. Blots with different exposure time were shown. The middle panel in the blot with 5 sec exposure was cropped to generate Fig 5D.

Supplementary Figure S3

Unprocessed Fig 7A

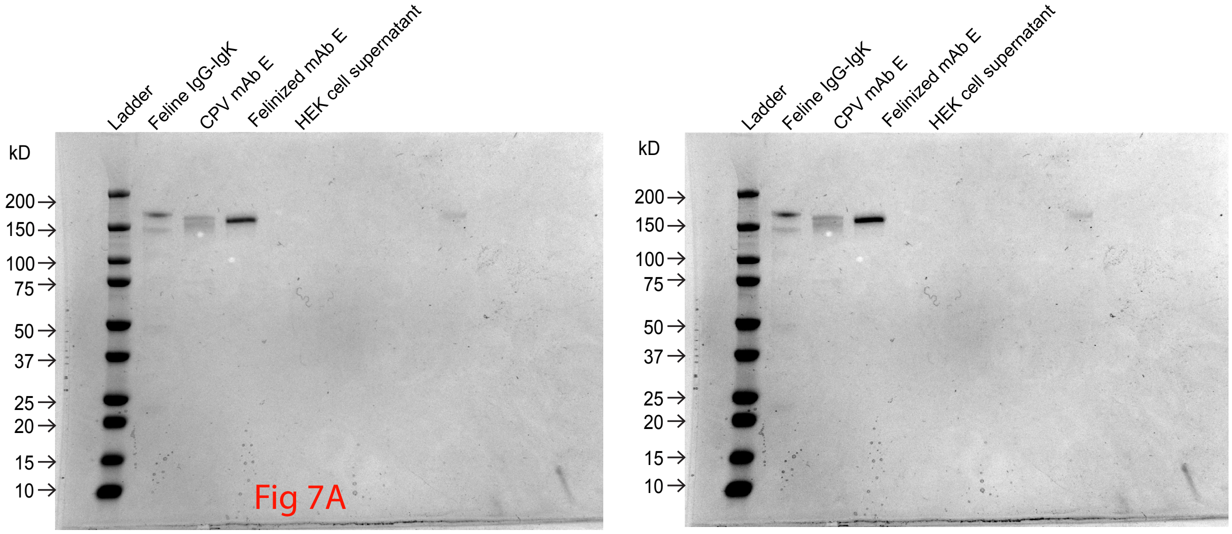

Full-length SDS-PAGE gel of Fig 7A as dectected by Coomassie staining autoexposure. The left gel was autoexposed to detect faint bands. The right gel was autoexposed to detect intense bands. The left gel was cropped to generate Fig 7A.

Unprocessed Fig 7B

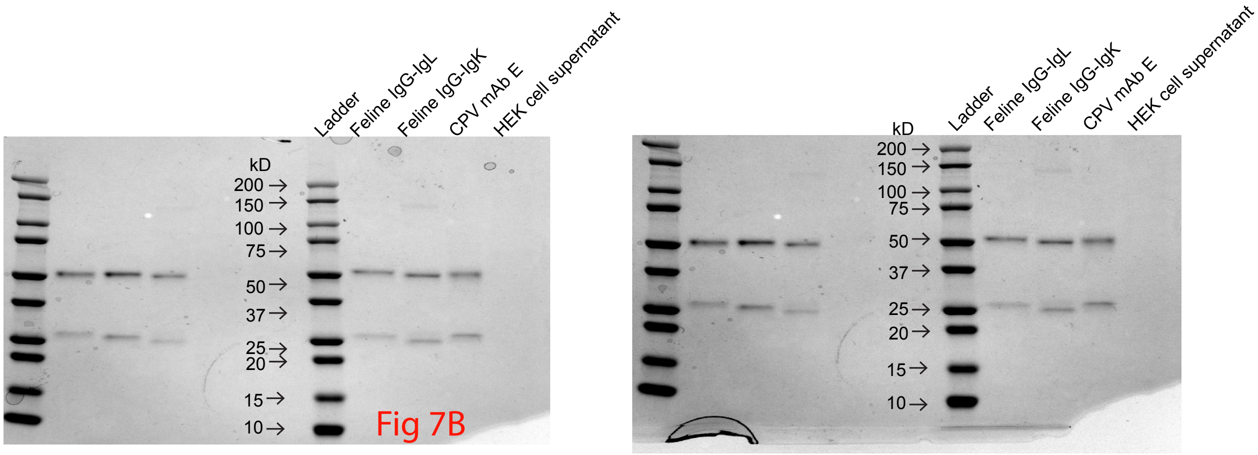

Full-length SDS-PAGE gel of Fig 7B as dectected by Coomassie staining autoexposure. The left gel was autoexposed to detect intense bands. The right gel was autoexposed to detect faint bands. The left rignt pannel was cropped to generate Fig 7B.

Unprocessed Fig 7C

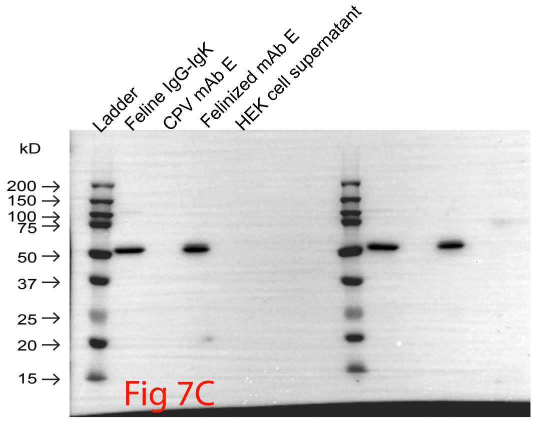

0.2 sec exposure

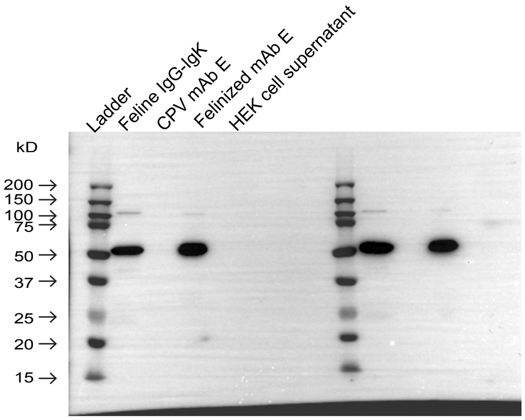

1 sec exposure

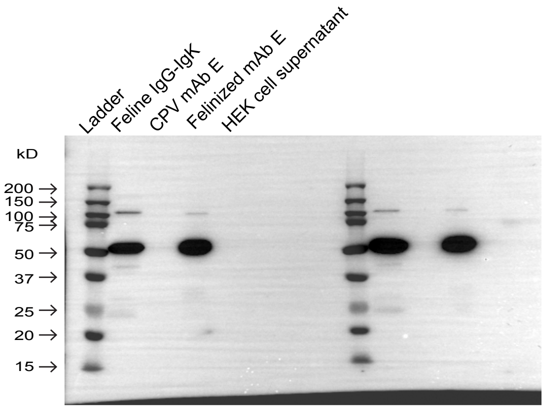

2 sec exposure

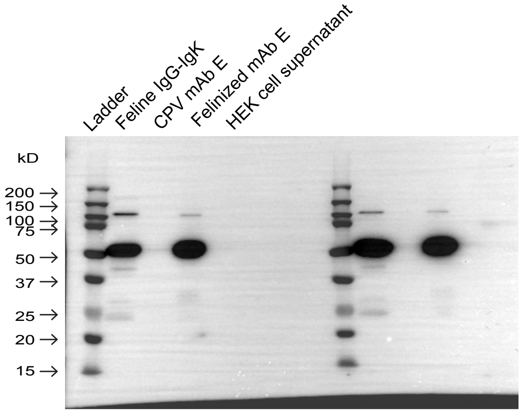

3 sec exposure

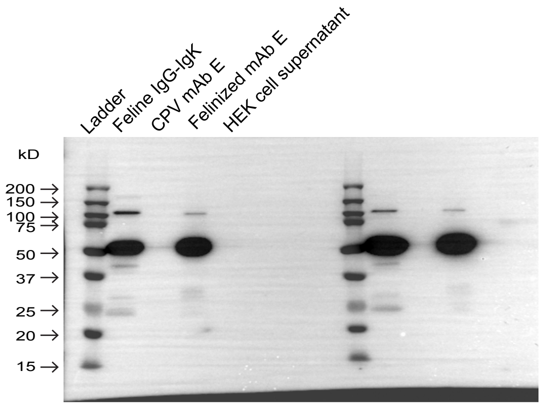

4 sec exposure

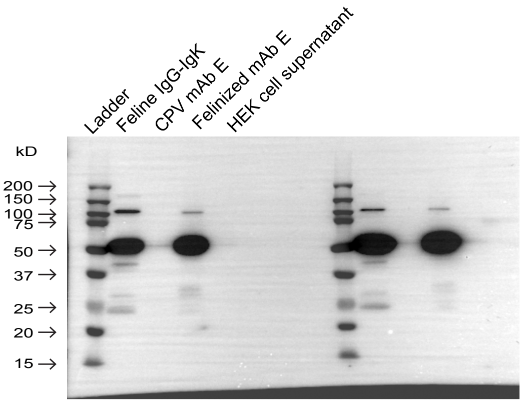

5 sec exposure

Full-length immunoblot of Fig 7C as dectected by goat-a-cat-HRP conjugated antibody. Blots with different exposure time were shown. The left panel of the blot with 0.2 sec exposure was cropped to generate Fig 7C.

Unprocessed Fig 7D

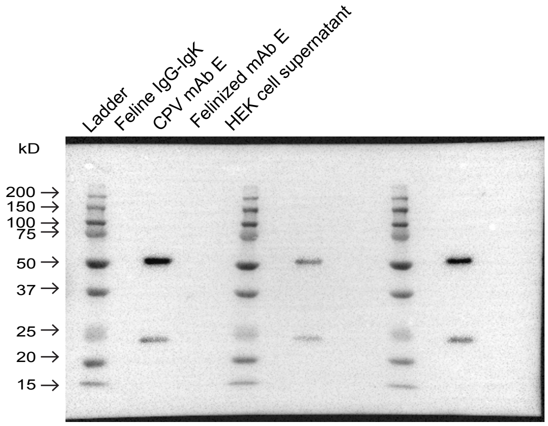

0.2 sec exposure

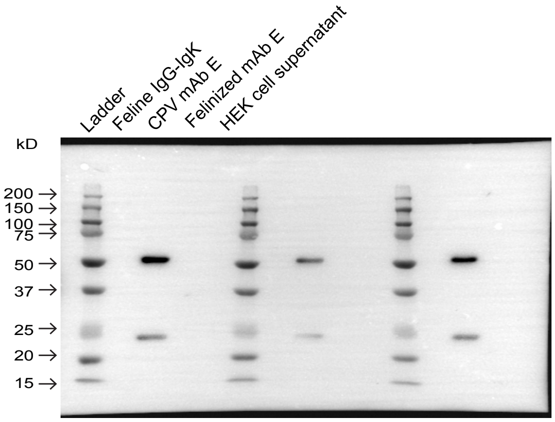

5 sec exposure

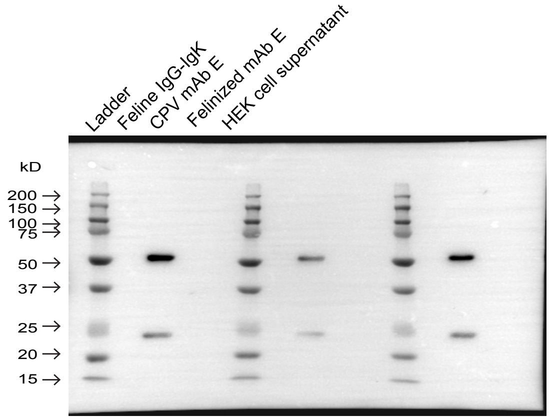

7.2 sec Autoexposure

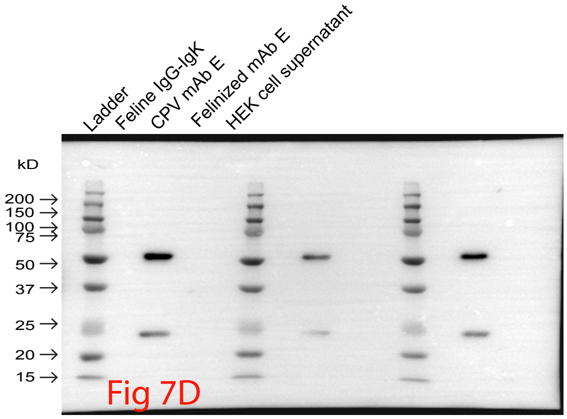

8 sec exposure

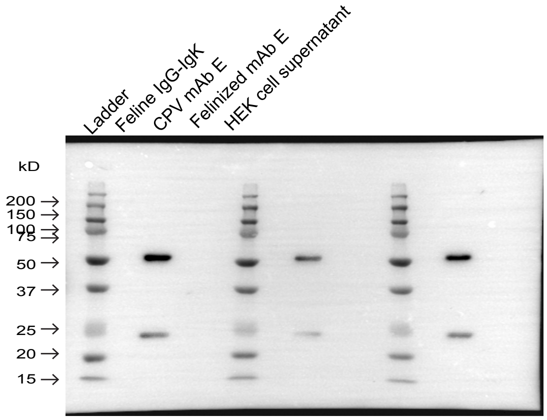

9 sec exposure

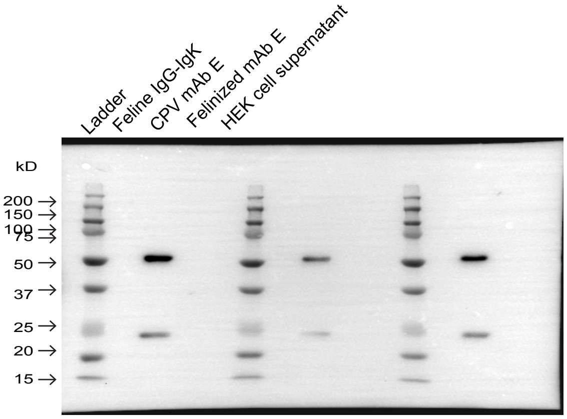

10 sec exposure

Full-length immunoblot of Fig 7D as dectected by goat-a-rat-HRP conjugated antibody. Blots with different exposure time were shown. The left panel of the blot with 8 sec exposure was cropped to generate Fig 7D.
